# Cardiac Tamponade as an Initial Manifestation of Cervical Cancer

**DOI:** 10.1155/2019/7524797

**Published:** 2019-01-09

**Authors:** Yuridia Evangelina Rodríguez-Rosales, Carlos Eduardo Salazar-Mejía, Blanca Angélica Soto-Martínez, David Hernández-Barajas, Oscar Vidal-Gutiérrez, Gabriela Sofia Gómez-Macías

**Affiliations:** ^1^Universidad Autónoma de Nuevo Leon, Facultad de Medicina y Hospital Universitario “Dr. José Eleuterio González”, Department of Internal Medicine, Av. Madero y Gonzalitos s/n, Colonia Mitras Centro, Monterrey, Nuevo Leon, C.P. 64460, Mexico; ^2^Universidad Autónoma de Nuevo Leon, Facultad de Medicina y Hospital Universitario “Dr. José Eleuterio González”, Centro Universitario Contra el Cáncer, Av. Madero y Gonzalitos s/n, Colonia Mitras Centro, Monterrey, Nuevo Leon, C.P. 64460, Mexico; ^3^Universidad Autónoma de Nuevo Leon, Facultad de Medicina y Hospital Universitario “Dr. José Eleuterio González”, Department of Pathology, Av. Madero y Gonzalitos s/n, Colonia Mitras Centro, Monterrey, Nuevo Leon, C.P. 64460, Mexico

## Abstract

Cervical cancer is the second most common malignancy worldwide in women and the third most common cause of cancer death in developing countries. This type of cancer spreads mainly to the lung, the bone, and the brain; however, the pericardium is an unusual site of invasion, which is associated with a poor prognosis. We present a case of a 35-year-old woman with six months of leg edema and abnormal uterine bleeding. During the initial evaluation, cardiac tamponade and a bilateral pleural effusion were found. A left supraclavicular lymphadenopathy was identified on physical examination, while gynecological examination and MRI were irrelevant. Initial cytology of the pericardial fluid showed a poorly differentiated carcinoma, and a cervical biopsy revealed a squamous cell invasive carcinoma. Chemotherapy was started with carboplatin and paclitaxel, but no clinical improvement was noted and the patient died 46 days after arrival. Cardiac tamponade in a young female patient is a harbinger to widen the differential diagnosis to include not only infectious, cardiac, or metabolic etiology but also oncological causes since this will allow appropriate treatment.

## 1. Introduction

Pericardial metastasis is an unusual manifestation of cervical cancer, generally identified at autopsy [1]. Symptomatic pericardial effusion and cardiac tamponade are usually described in the scenario of recurrent disease after previous treatment with chemotherapy and/or radiotherapy, with very few cases reporting these entities as an initial presentation of cervical cancer [2]. Herein, we present a case of cardiac tamponade as an initial manifestation of a squamous cell carcinoma of the cervix.

## 2. Case Report

**Figure 1:**
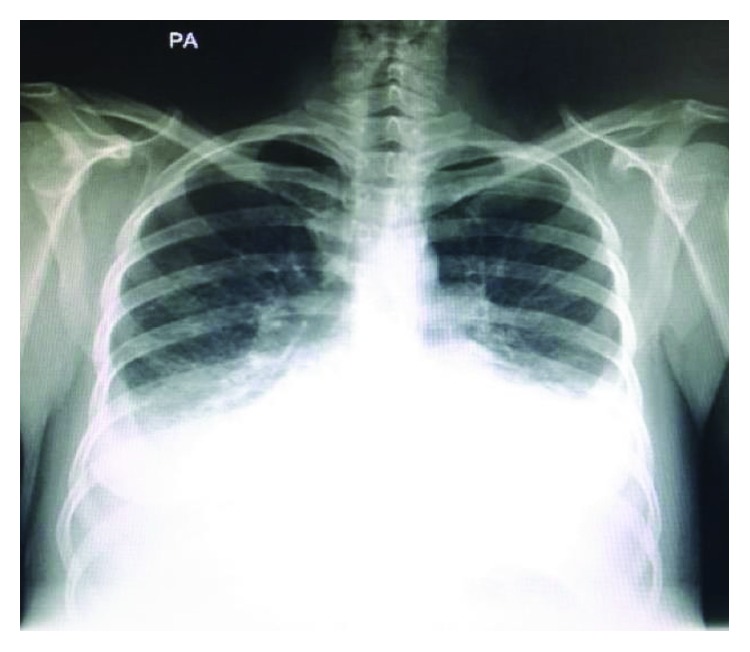
Chest X-ray.

A 35-year-old woman arrived at the emergency department because of rest dyspnea and a 6-month history of lower extremity edema. She had a 3-month history of intermittent abnormal vaginal bleeding. On initial evaluation, the patient was hypoxemic with an oxygen saturation of 80% with room air. Relevant clinical signs were tachycardia and hypotension, decreased heart sounds, and a left supraclavicular lymphadenopathy. A chest X-ray showed a widening of the cardiac silhouette with a bilateral pleural effusion (Figure 1).

**Figure 2:**
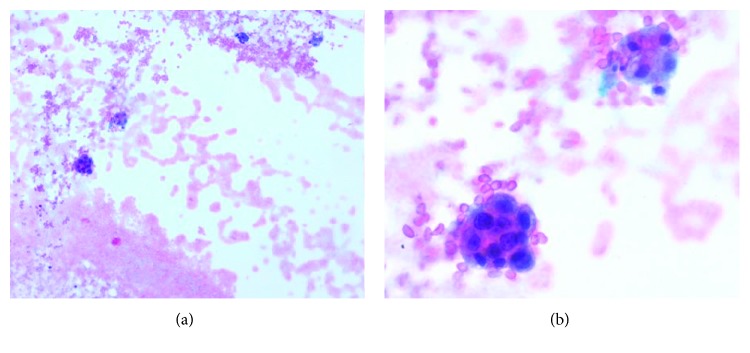
(a) Pleural and pericardial fluid cytology (10x) shows mesothelial cells with hyperplasia; the second population of cells are malignant squamous epithelial cells. (b) Pleural and pericardial fluid cytology (40x). A close-up of mesothelial cells; a group of malignant squamous cells is seen in the lower part of the image.

Pericardiocentesis was performed and a total of 500 mL of bloody secretion was drained with symptomatic improvement. Pleural fluid was obtained by thoracocentesis, and cytology was positive for a poorly differentiated carcinoma (Figure 2).

**Figure 3:**
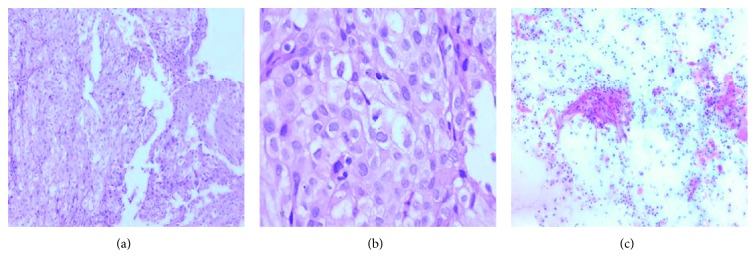
(a) Cervical biopsy, 5x, invasive nonkeratinized squamous cell; (b) intercellular bridge, nuclear hyperchromia, macronucleolus, and atypical mitosis, 40x; and (c) cervical cytology with invasive squamous cell carcinoma.

**Figure 4:**
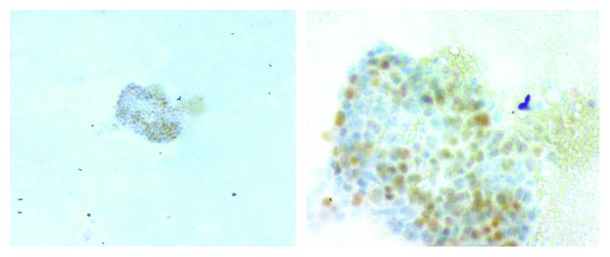
Immunochemistry, P63(+); immunophenotype for malignant squamous cells.

An excisional biopsy of the left supraclavicular lymphadenopathy was positive for metastatic squamous cell carcinoma. The cervical biopsy reported a squamous cell carcinoma associated with an intraepithelial high-grade lesion (Figures 3 and 4). CA-125 was 335.5 IU/mL and a simple and contrasted pelvic MRI demonstrated a uterine and cervical absence of tumoral mass; however, peritoneal carcinomatosis was present.

Chemotherapy was begun with carboplatin and paclitaxel. Despite the treatment received during her hospitalization, she again presented a pericardial and pleural effusion with subsequent hemodynamic instability and respiratory failure. Due to the fact that in our center there is no experience in applying intrapericardial sclerotherapy, it was offered to repeat pericardiocentesis; however, this intervention was refused. The patient died 46 days after the initial presentation.

## 3. Discussion

Cervical cancer is the second most common cancer diagnosed in women worldwide and the third cause of cancer death in developing countries [1, 3, 4]. The main sites for metastasis are the lung, the bone, and the brain [2]. Metastasis to the pericardial sac is an unusual manifestation. It has a reported incidence of 1.2-7% [2, 5, 6], conferring a poor prognosis with an overall survival of 2 to 5 months from diagnosis [2], with the majority of cases discovered at autopsy [7–10]. To our knowledge, this is the first case of cardiac tamponade as the initial presentation of a squamous cell carcinoma of the cervix.

The most common causes of pericardial effusion with or without tamponade are infections (*Coxsackievirus*, VEB, CMV, and *M. tuberculosis*); autoimmune diseases; cancer from lymphatic or hematogenous dissemination (metastasis: melanoma (50%), lung (30%), breast (12%), and lymphoma (12%)) [5, 9, 11, 12]; cardiac diseases (Dressler syndrome, myocarditis, and aortic dissection aneurysm); trauma; metabolic diseases (hypothyroidism, uremia, and ovary hyperstimulation); or drugs (cyclophosphamide, doxorubicin, gemcitabine, cytarabine, fludarabine, docetaxel, isoniazid, hydralazine, and phenytoin) [1, 13].

Maisch et al. analyzed 357 pericardial effusion samples from 1988 to 2008 and identified 68 patients with cancer-associated pericardial effusion. In 42 patients, a malignant pericardial effusion was noted; in 15 patients, it was induced by radiation; in 11, by viral disease; and in 6, with an autoimmune process. From the cancer-associated pericardial effusion, it was found that 52.4% was from lung cancer, 19% breast cancer, 4.8% Hodgkin's lymphoma, 4.8% colon cancer, 2.4% mesothelioma and esophageal cancer, and 14.2% was of unknown origin undifferentiated cancer [14].

Pericardial effusion as a clinical presentation can be acute (trauma, aortic rupture, and iatrogenic), subacute (uremia or idiopathic), or chronic (constrictive or adhesive). The clinical features are dyspnea, pleuritic pain, cough, fatigue, and syncope. Cardiac tamponade causes hypotension, tachycardia, and decreased heart sounds (Beck triad). The paradoxical pulse is reported as the most sensitive sign (82%) to diagnose cardiac tamponade, followed by tachycardia and elevated jugular venous pressure with a sensitivity of 77% and 76%, respectively [1, 5, 13]. From the initial evaluation, the widening of the cardiac silhouette can be associated with the “water bottle sign” and the concomitant bilateral pleural effusion. The EKG demonstrates low-voltage QRS and nonspecific T wave and ST segment changes. A transthoracic echocardiogram helps assess size, location, and hemodynamic physiology [1].

Pericardiocentesis is a diagnostic and therapeutic procedure. The drainage of the pericardial fluid is assessed daily. The inserted catheter is removed when drainage is less than 30 mL/day. Such a procedure has a greater risk of major complication (1.2%) such as ventricle laceration, pneumothorax, ventricular tachycardia, and bacteremia. In patients with cancer, the risk of recurrence is about 90% [1, 13]. There are many treatment options for pericardial effusion recurrence such as the use of an indwelling catheter with an efficacy of 70-90%, a pericardial window with drainage to the pleural or peritoneal cavity (recurrence of 5-15%), or pericardial sclerosis with chemotherapeutic agents such as cisplatin, bleomycin, or tetracycline [12].

Table 1 summarizes the reported literature regarding cervical cancer associated with pericardial effusion and cardiac tamponade. The mean age for diagnosis was 52 years. Cardiac tamponade was reported with pericardial effusion 6.2 months after the initial diagnosis and mostly in patients with previous treatment. Pericardial tamponade was detected in one patient 5 days after cervical cancer diagnosis with an overall survival of 4 months after pericardiocentesis [9]. Also, Azria et al., in 2011, published a similar case of a 54-year-old woman who initially presented cardiac tamponade, which was posteriorly associated with metastatic cervical adenocarcinoma and who died 33 days after its diagnosis [20].

Within the initial approach of a young woman presenting with cardiac tamponade, an etiology must be identified and cancer should be considered as a possible cause. A correct workup is required to achieve a timely diagnosis, in order to grant the patient the best possible outcome.

## Figures and Tables

**Table 1:** Reported cases of cervical cancer with pericardial effusion and cardiac tamponade.

Author (year)	Age at initial presentation (years)	Time from diagnosis to pericardial effusion	FIGO clinical stage (initial)	Previous treatment	Presence of cardiac tamponade	Treatment after diagnosis of pericardial effusion	Overall survival
Charles et al. (1997) [15]	46	24 months	IIIB	RT, hysterectomy+BSO	Yes	Pericardial window, CT, doxorubicin	8 months
Rieke and Kapp (1988) [5]	49	23 months	IIA	Hysterectomy+BSO, RT	No	Pericardiocentesis, RT	9 months
Rudoff et al. (1989) [16]	27	21 months	IIIB	RT	Yes	Pericardiocentesis, anterior pericardiectomy, cisplatinum	Not reported
Nelson and Rose (1993) [9]	51	5 days	IV	None	Yes	Pericardiocentesis/CT cisplatin+RT	4 months
	61	3 months	IIIB	RT	Yes	Pericardiocentesis, instillation of tetracycline, CT cisplatin, bleomycin, methotrexate alternating with cisplatin and 5FU	12 months
Kountz et al. (1993) [17]	28	10 months	IIB	RT/CT	No/mass in right ventricle	Not specified	3 months
Jamshed et al. (1996) [6]	57	32 months	IB	Hysterectomy, RT	Yes	Pericardiocentesis, pericardial window, RT	5 months
Lemus et al. (1998) [10]	53	24 months	IB	RT, hysterectomy+BSO+superior vaginectomy	No/interventricular septum mass	RT	1 month
	49	12 months	IVB	RT	No/mass in right ventricle	CT 5FU+cisplatin	7 months
Senzaki et al. (1999) [18]	28	16 months		Hysterectomy, RT/CT	No/mass in right ventricle	Pericardiocentesis+intrapericardial cisplatinum	1 month
Kim et al. (2008) [19]	64	6 months	IB	CT carboplatin+paclitaxel+concurrent RT pre- and posthysterectomy	No/right atrium mass	5-fluorouracil+cisplatin+RT	12 months
Kim et al. (2011) [1]	52	6 months	IVB	3 cycles of 5FU, cisplatin+concurrent RT	Yes	Pericardiocentesis	1 month
Azria et al. (2011) [20]	54	Initial presentation (cervical adenocarcinoma)	IVB	None	Yes	Pericardiocentesis, pericardial window, carboplatin+paclitaxel	33 days
Ore et al. (2013) [21]	5th decade	9 months	IVB	RT, CT topotecan+cisplatin	Yes	Pericardiocentesis, pericardial window	26 days
Kalra et al. (2014) [2]	56	6 months	IIIB	Carboplatin+paclitaxel+RT	Yes	CT not specified+RT	Not reported
Ramegowda et al. (2015) [11]	50	23 months	IIIB	RT, brachytherapy	Yes	No treatment	4 months
Tsuchida et al. (2016) [22]	78	15 months	IIIB	RT	No/mass in right ventricle	No treatment	1 month

FIGO: International Federation of Gynecology and Obstetrics; CT: chemotherapy; RT: radiotherapy; BSO: bilateral salpingo-oophorectomy; 5FU: 5-fluorouracil.
